# FLIPPING STIGMA ON ITS EAR: A TOOLKIT FROM PARTICIPATORY ACTION RESEARCH

**DOI:** 10.1093/geroni/igac059.1582

**Published:** 2022-12-20

**Authors:** Mariko Sakamoto, Jim Mann, Deborah O'Connor, Alison Phinney

**Affiliations:** University of British Columbia, Vancouver, British Columbia, Canada; University of British Columbia, Vancouver, British Columbia, Canada; School of Social Work - University of British Columbia, Vancouver, British Columbia, Canada; University of British Columbia, Vancouver, British Columbia, Canada

## Abstract

People living with dementia face persistent stigma, discrimination and social exclusion, with significant emotional, physical and social consequences. Addressing this requires changing attitudes and fostering actions for communities to include people with dementia as citizens with agency and self-determination. This presentation highlights the work of an Action Group (AG) of people living with dementia. As part of a four-year Participatory Action Research study aimed at addressing the stigma, discrimination and social exclusion that is so common to the dementia experience, members of the AG in partnership with the research team developed the Flipping Stigma on its Ear Toolkit. Focus will be on the action-oriented nature of this research project, an overview of the toolkit, and exploration of the communal space that was created by AG members in the process of working together, learning from one another, and making a collective contribution towards addressing stigma, discrimination and social exclusion.

